# Supplementary material for: Membranous nephropathy in the UK Biobank
Source: PLoS One. 2023 Apr 27;18(4):e0281795. doi: 10.1371/journal.pone.0281795 (PMC10138203; doi:10.1371/journal.pone.0281795)
Supplement: S1 Fig — B) Age distributions of the cases identified in the two sources. C) Date of diagnosis of the cases identified in the two sources. (PDF) [file pone.0281795.s001.pdf]

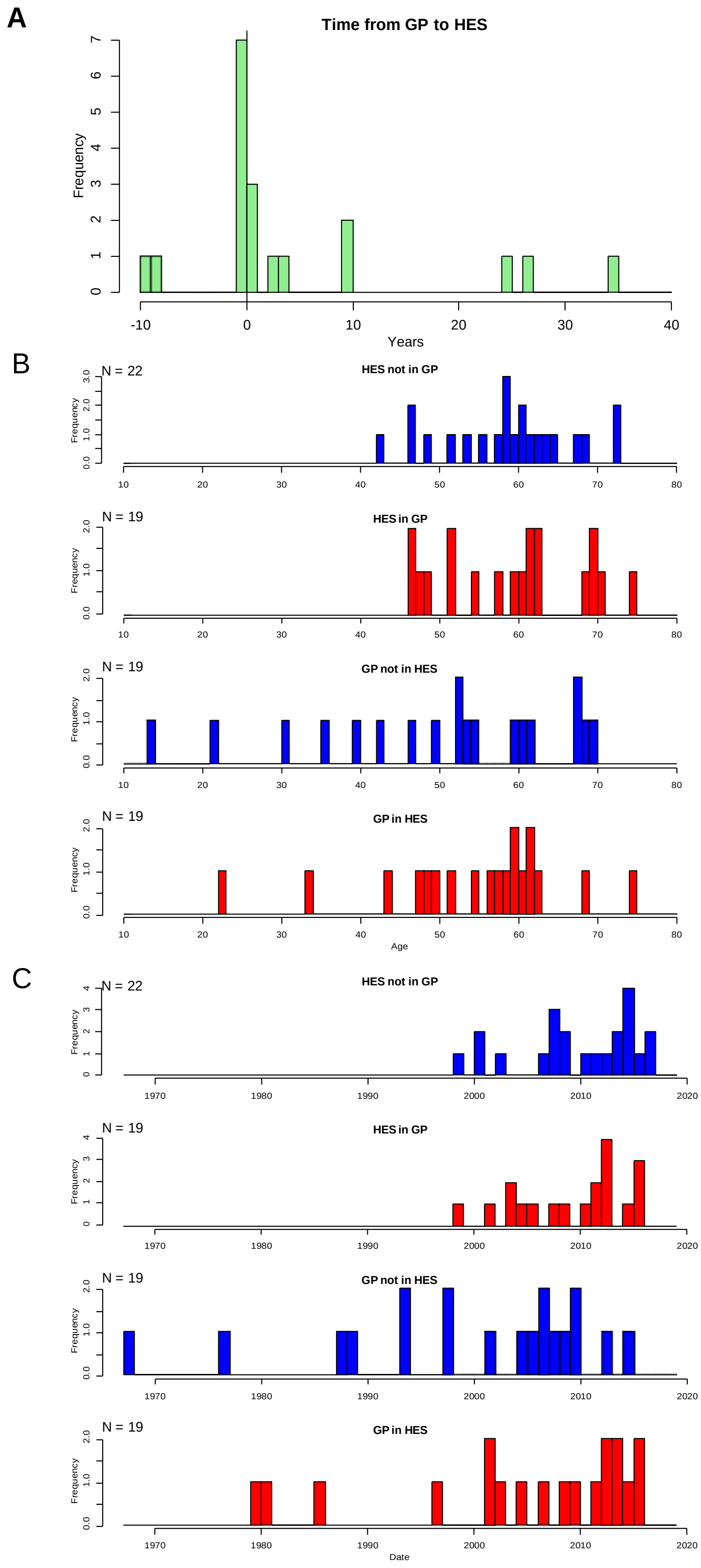

Figure S7: A) Time difference from GP diagnosis to HES diagnosis where the case was identified in both sources. B) Age distributions of the cases identified in the two sources. C) Date of diagnosis of the cases identified in the two sources.
