# Supplementary material for: Membranous nephropathy in the UK Biobank
Source: PLoS One. 2023 Apr 27;18(4):e0281795. doi: 10.1371/journal.pone.0281795 (PMC10138203; doi:10.1371/journal.pone.0281795)
Supplement: S1 Table — (PDF) [file pone.0281795.s002.pdf]

	Description	Details	Biobank Field
Table 2	Age		f.21003.0.0
	Sex		f.31.0.0
	Ethnicity		f.21000.0.0
	BMI		f.21001.0.0
	Smoking	Current smoker	f.20116.0.0
	Alcohol		f.20117.0.0
	Alcohol Frequency		f.1558.0.0
	Townsend Deprivation		f.189.0.0
	IMD		f.26410.0.0 | f.26426.0.0 | f.26427.0.0 (Three fields, use later ones if earlier ones missing)
Tables 2,3	N022	HES data: ICD code in any of primary or secondary diagnosis codes	f.41202,f.41204: ICD code in either field
	N032		
	N042		
	N052		
	Putative MN	Any of N022,N032,N042,NO052	
	CKD	Any N18	
	MN diagnosis date	HES	HES data: visit date of first event
	Follow-up Time	(Last HES refresh date|date of death)-baseline visit date	min(2019-03-01,f.40000.0.0) - f.53.0.0
Tables 4,5, S2, S3, S5	HLADQ1 (GWAS1)	rs2187668 Ch 6	Relevant SNPs extracted from the bulk genetic data (see methods)
	PLA2R1 (GWAS1)	rs4664308 Ch 2	
	HLADQ1 (GWAS2)	rs9272729 Ch 6	
	PLA2R1 (GWAS2)	rs17830558 Ch 2	
	HLADRB1/DQA1 (GWAS3)	rs9271573	
	PLA2R1 (GWAS3)	rs17831251	
	NFKB1 (GWAS3)	rs230540	
	IRF4 (GWAS3)	rs9405192	
Table 6	NO2: 2005		f.24016.0.0
	NO2: 2006		f.24017.0.0
	NO2: 2007		f.24018.0.0
	NO2: 2010		f.24003.0.0
	NO: 2010		f.24004.0.0
	PM: <2.5		f.24006.0.0
	PM: 2.5-10		f.24008.0.0
	PM >10		f.24005.0.0
	Distance to main road	reciprocal of Biobank variable	f.24012.0.0
Tables 3, S4, S5	Self-reported Renal Failure	Self-report	f.20002.0 contains 1192|1193|1194
	HES CKD		f.41202,f.41204 HES ICD code N18
	HES Renal Disease		f.41202,f.41204 HES ICD code N0*,N1*
	Algorithmically derived ESRD		f.42026.0.0
	uACR	Albumin/Creatinine	f.30500.0.0,f.30510.0.0
	eGFR		computed from above and demographic data
	Self-reported Diabetes	Self-report	f.20002.0 contains 1222|1223|1521
	HES Diabetes	HES	f.41202;f.41204 HES ICD codes E10,E11,E12,E13,E14

	Self Report Coeliac	Self-report	f.20002.0 contains 1456
	HES Coeliac	HES	HES ICD code = K900
	Self-reported RenalBiopsy	Self-report	Any f.20004.0.*=1618
	OPCS RenalBiopsy	HES	HES operation codes: M08.1,M11.1,M11.2,M11.3,M11.8,M11.9,M13.1
Table S6	Vapours		Derived from occupation exposure matrix and SOC codes in f.22617.0.0 (see methods)
	Gases		
	Dusts		
	Biological Dusts		
	Mineral Dusts		
	Fumes		
	Diesel		
	Fibres		
	Mists		
	Asthmagens		
	Metals		
	Gases or Fumes		
	VGDF		
	VGDFFM		
Figure S7	Date of GP diagnosis		Derived from dates of GP records with the relevant Read2/3 codes for putative MN (see methods)

Table S1 Biobank fields and variable definitions
