# Supplementary material for: Membranous nephropathy in the UK Biobank
Source: PLoS One. 2023 Apr 27;18(4):e0281795. doi: 10.1371/journal.pone.0281795 (PMC10138203; doi:10.1371/journal.pone.0281795)
Supplement: S2 Table — (PDF) [file pone.0281795.s003.pdf]

			Allele Frequency (%)
GWAS1	HLADQA1 rs2187668	CC	357516/487208 (73.4%)
		CT	118070/487208 (24.2%)
		TT	11622/487208 (2.4%)
		MAF	14.5%
	PLA2R1 rs4664308	AA	174588/487208 (35.8%)
		AG	230759/487208 (47.4%)
		GG	81861/487208 (16.8%)
		MAF	40.5%
GWAS2	HLADQA1 rs9272729	GG	351417/479895 (73.2%)
		GA	117731/479895 (24.5%)
		AA	10747/479895 (2.2%)
		MAF	14.5%
	PLA2R1 rs17830558	TT	105620/472780 (22.3%)
		TG	228842/472780 (48.4%)
		GG	138318/472780 (29.3%)
		MAF	46.5%
GWAS3	HLADRB1/DQA1 rs9271573	CC	170192/486978 (34.9%)
		AC	234411/486978 (48.1%)
		AA	82375/486978 (16.9%)
		MAF	41%
	PLA2R1 rs17831251	CC	172495/481109 (35.9%)
		CT	227843/481109 (47.4%)
		TT	80771/481109 (16.8%)
		MAF	40.5%
	NFKB1 rs230540	TT	205265/481333 (42.6%)
		CT	216576/481333 (45.0%)
		CC	59492/481333 (12.4%)
		MAF	34.9%
	IRF4 rs9405192	GG	259096/474386 (54.6%)
		GA	182247/474386 (38.4%)
		AA	33043/474386 (7.0%)
		MAF	26.2%

Table S2 – Allele counts at the lead SNPs from GWAS1[4], GWAS2[5], and GWAS3[6] in all UK Biobank participants using the imputed UK Biobank datasets. GWAS – Genome-Wide Association Study, MAF – minor allele frequency, SNP – single nucleotide polymorphism
