# Supplementary material for: Membranous nephropathy in the UK Biobank
Source: PLoS One. 2023 Apr 27;18(4):e0281795. doi: 10.1371/journal.pone.0281795 (PMC10138203; doi:10.1371/journal.pone.0281795)
Supplement: S3 Table — (PDF) [file pone.0281795.s004.pdf]

		GWAS1 PLA2R1 rs4664308		
		AA	AG	GG
GWAS1 HLADQA1 rs2187668	CC	129287 (26.5%)	168473 (34.6%)	59756 (12.3%)
	CT	41222 (8.5%)	56717 (11.6%)	20131 (4.1%)
	TT	4079 (0.8%)	5569 (1.1%)	1974 (0.4%)
		GWAS2 PLA2R1 rs17830558		
		GG	TG	TT
GWAS2 HLADQA1 rs9272729	AA	3167 (0.7%)	5042 (1.1%)	2230 (0.5%)
	GA	33948 (7.3%)	56205 (12.1%)	24352 (5.2%)
	GG	99131 (21.3%)	164081 (35.2%)	77518 (16.6%)

Table S3 – Counts of genotype combinations at the lead SNPs from GWAS1[4] and GWAS2[5] in all UK Biobank participants using both the imputed UK Biobank datasets. GWAS – Genome-Wide Association Study, SNP – single nucleotide polymorphism
