# Supplementary material for: Membranous nephropathy in the UK Biobank
Source: PLoS One. 2023 Apr 27;18(4):e0281795. doi: 10.1371/journal.pone.0281795 (PMC10138203; doi:10.1371/journal.pone.0281795)
Supplement: S4 Table — (PDF) [file pone.0281795.s005.pdf]

		Low or medium risk genotype<br>No TTAA	High risk genotype<br>TTAA
		N= 498,428	N= 4,079
Self-reported Kidney Failure		878/498428 (0.2%)	6/4079 (0.1%)
HES CKD		9003/498428 (1.8%)	79/4079 (1.9%)
HES Kidney Disease		21826/498428 (4.4%)	205/4079 (5.0%)
Algorithmically derived ESKD		1184/498428 (0.2%)	7/4079 (0.2%)
uACR at baseline (mg/mmol)	<3	454866/480358 (94.7%)	3717/3936 (94.4%)
	3-30	23237/480358 (4.8%)	191/3936 (4.9%)
	30-250	2118/480358 (0.4%)	25/3936 (0.6%)
	>250	137/480358 (0.03%)	3/3936 (0.08%)
	Median (IQR)	0.4 (0.4-0.6)	0.4 (0.4-0.6)
	Unknown	18070	143
eGFR at baseline (ml/min/1.73m^2)	≥90	275085/464974 (59.2%)	2288/3896 (58.7%)
	60-90	179252/464974 (38.6%)	1508/3896 (38.7%)
	15-60	10496/464974 (2.3%)	99/3896 (2.5%)
	<15	141/464974 (0.03%)	1/3896 (0.03%)
	Median (IQR)	92.8 (82.9-100.1) [missing]	92.7 (82.6-100.3) [missing]
	Unknown	33454	183
Self-reported Diabetes		25220/498428 (5.1%)	260/4079 (6.4%)
HES Diabetes		30975/498428 (6.2%)	308/4079 (7.6%)
Self-reported Coeliac Disease		1934/498428 (0.4%)	115/4079 (2.8%)
HES Coeliac Disease		2351/498428 (0.5%)	131/4079 (3.2%)
Self-reported Kidney Biopsy		129/498428 (0.03%)	3/4079 (0.07%)
OPCS Kidney Biopsy		292/410203 (0.07%)	2/3414 (0.06%)

Table S4 – Phenotype per combined genotype group at HLADQA1 (rs2187668, test allele T) and PLA2R1 (rs4664308, test allele A) from GWAS1[4] in all UK Biobank participants using the directly genotyped UK Biobank dataset. MN = Membranous Nephropathy, HES = Hospital Episode Statistics, CKD = Chronic Kidney Disease, ESKD = End-Stage Kidney Disease, ACR = urinary albumin:creatinine ratio, eGFR = estimated Glomerular Filtration Rate, IQR = Inter-Quartile Range, SD = Standard Deviation, OPCS = OPCS Classification of Interventions and Procedures
