# Supplementary material for: Membranous nephropathy in the UK Biobank
Source: PLoS One. 2023 Apr 27;18(4):e0281795. doi: 10.1371/journal.pone.0281795 (PMC10138203; doi:10.1371/journal.pone.0281795)
Supplement: S5 Table — (PDF) [file pone.0281795.s006.pdf]

ACR > 3 mg/mmol prevalence		N (%)	RR
Kidney Failure	No	25360/483509 (5.2%)	1
	Yes	351/785 (44.7%)	8.5 (7.9-9.2)
Diabetes	No	21491/459995 (4.7%)	1
	Yes	4220/24299 (17.4%)	3.7 (3.6-3.8)
Diabetes / Kidney Failure combinations	No/No	21240/459377 (4.6%)	1
	Yes/No	4120/24132 (17.1%)	3.7 (3.6-3.8)
	No/Yes	251/618 (40.6%)	8.8 (8.0-9.7)
	Yes/Yes	100/167 (59.9%)	13 (11.4-14.7)
ACR >30 mg/mmol prevalence			
Kidney Failure	No	2134/483509 (0.4%)	1
	Yes	149/785 (19.0%)	43 (37-50)
Diabetes	No	1652/459995 (0.4%)	1
	Yes	631/24299 (2.6%)	7.2 (6.6-7.9)
Diabetes / Kidney Failure combinations	No/No	1552/459377 (0.3%)	1
	Yes/No	582/24132 (2.4%)	7.1 (6.5-7.8)
	No/Yes	100/618 (16.2%)	47.9 (39.8-57.7)
	Yes/Yes	49/167 (29.3%)	86.8 (68.3-110.5)
ACR >3 mg/mmol prevalence			
GWAS1 HLADQA1 rs2187668	CC	18563/347004 (5.3%)	1
	CT	5852/114580 (5.1%)	0.95 (0.93-0.98)
	TT	563/11243 (5.0%)	0.94 (0.86-1.02)
GWAS1 PLA2R1 rs4664308	GG	4142/79512 (5.2%)	1
	AG	11612/224105 (5.2%)	0.99 (0.96-1.03)
	AA	9224/169210 (5.5%)	1.05 (1.01-1.08)
GWAS2 HLADQA1 rs9272729	GG	18242/341085 (5.3%)	1
	AG	5831/114258 (5.1%)	0.95 (0.93-0.98)
	AA	520/10396 (5.0%)	0.94 (0.86-1.02)
GWAS2 PLA2R1 rs17830558	GG	6953/134353 (5.2%)	1
	AG	11514/222065 (5.2%)	1 (0.97-1.03)
	AA	5761/102396 (5.6%)	1.09 (1.05-1.12)
ACR >30 mg/mmol prevalence			
GWAS1 HLADQA1 rs2187668	CC	1621/347004 (0.5%)	1
	CT	529/114580 (0.5%)	0.99 (0.9-1.09)
	TT	61/11243 (0.5%)	1.16 (0.9-1.5)
GWAS1 PLA2R1 rs4664308	GG	358/79512 (0.5%)	1
	AG	1004/224105 (0.4%)	1 (0.88-1.12)
	AA	849/169210 (0.5%)	1.11 (0.99-1.26)
GWAS2 HLADQA1 rs9272729	GG	1596/341085 (0.5%)	1
	AG	532/114258 (0.5%)	1 (0.9-1.1)
	AA	55/10396 (0.5%)	1.13 (0.86-1.48)
GWAS2 PLA2R1 rs17830558	GG	607/134353 (0.5%)	1
	AG	1012/222065 (0.5%)	1.01 (0.91-1.12)
	AA	520/102396 (0.5%)	1.12 (1-1.26)

Table S5 – Phenotype and genotype by degree of proteinuria for all UK Biobank participants, with genotypes obtained from the imputed UK Biobank dataset at the lead SNPs from GWAS1[4] and GWAS2[5]. GWAS – Genome-Wide Association Study, SNP – single nucleotide polymorphism, ACR = urinary albumin:creatinine ratio, RR – Relative Risk
