# Supplementary material for: Membranous nephropathy in the UK Biobank
Source: PLoS One. 2023 Apr 27;18(4):e0281795. doi: 10.1371/journal.pone.0281795 (PMC10138203; doi:10.1371/journal.pone.0281795)
Supplement: S6 Table — (PDF) [file pone.0281795.s007.pdf]

Occupational Exposure		N (%)		RR (95% CI)
		No MN	MN	
Vapours	Not Exposed	264341 (91.3%)	44 (86.3%)	1
	Exposed	60666 (18.7%)	7 (13.7%)	0.693 (0.312-1.539]
Gases	Not Exposed	277742 (85.5%)	46 (90.2%)	1
	Exposed	47265 (14.5%)	5 (9.8%)	0.639 (0.254-1.607]
Dusts	Not Exposed	237450 (73.1%)	39 (76.5%)	1
	Exposed	87557 (26.9%)	12 (23.5%)	0.834 (0.437-1.594]
Biological Dusts	Not Exposed	278703 (85.8%)	44 (86.3%)	1
	Exposed	46304 (14.2%)	7 (13.7%)	0.958 (0.431-2.126]
Mineral Dusts	Not Exposed	272337 (83.8%)	44 (86.3%)	1
	Exposed	52670 (16.2%)	7 (13.7%)	0.823 (0.371-1.826]
Fumes	Not Exposed	272639 (83.9%)	44 (86.3%)	1
	Exposed	52368 (16.1%)	7 (13.7%)	0.828 (0.373-1.839]
Diesel	Not Exposed	298495 (91.8%)	46 (90.2%)	1
	Exposed	26512 (8.2%)	5 (9.8%)	1.224 (0.486-3.079]
Fibres	Not Exposed	296119 (91.8%)	47 (92.2%)	1
	Exposed	28888 (8.9%)	4 (7.8%)	0.872 (0.314-2.421]
Mists	Not Exposed	282350 (86.9%)	46 (90.2%)	1
	Exposed	42657 (13.1%)	5 (9.8%)	0.719 (0.286-1.811]
Asthmagens	Not Exposed	255602 (78.6%)	42 (82.4%)	1
	Exposed	69405 (21.4%)	9 (7.6%)	0.789 (0.384-1.621]
Metals	Not Exposed	303918 (93.5%)	49 (96.1%)	1
	Exposed	21089 (6.5%)	2 (3.9%)	0.588 (0.143-2.419]
Gases or Fumes	Not Exposed	300798 (92.6%)	47 (92.2%)	1
	Exposed	24209 (7.4%)	4 (7.8%)	1.057 (0.381-2.935]
VGDF (Vapours, Gases, Dusts, or Fumes)	Not Exposed	215762 (66.4%)	35 (68.6%)	1
	Exposed	109245 (33.6%)	16 (31.4%)	0.903 (0.500-1.631]
VGDFFM (Any Exposures)	Not Exposed	215575 (66.3%)	35 (68.6%)	1
	Exposed	109432 (33.7%)	16 (31.4%)	0.901 (0.498-1.627]

Table S6 – environmental exposures – work-related
